# 2,2′-Methylenebis (6-tert-butyl 4-methylphenol) enhances the antitumor efficacy of belotecan, a derivative of camptothecin, by inducing autophagy

**DOI:** 10.18632/oncotarget.22858

**Published:** 2017-12-01

**Authors:** Minsu Jang, Hyunju Kim, Rackhyun Park, Daum Jo, Eun-Ju Lee, Won Keun Oh, Junsoo Park

**Affiliations:** ^1^ Division of Biological Science and Technology, Yonsei University, Wonju 26493, Republic of Korea; ^2^ Department of Obstetrics and Gynecology, Chung-Ang University School of Medicine, Seoul 06980, Republic of Korea; ^3^ Korea Bioactive Natural Material Bank, College of Pharmacy, Seoul National University, Seoul 08826, Republic of Korea

**Keywords:** autophagy, cancer, methylenebis, belotecan, camptothecin

## Abstract

Autophagy regulation is important for tumor cell survival. Activation and inhibition of autophagy can sensitize tumor cells to anticancer drugs. However, few autophagy-regulating small molecules are available to increase the efficacy of anticancer drugs. Here, we report that 2,2′-methylenebis (6-tert-butyl 4-methylphenol), hereafter referred to as methylenebis, is a novel autophagy-regulating small molecule that sensitizes tumor cells to belotecan, which is a derivative of camptothecin, a topoisomerase I inhibitor. Methylenebis activates autophagic flux by increasing the level of LC3-II and forming autolysosome puncta. Moreover, methylenebis enhances the antitumor efficacy of belotecan by activating both autophagy and apoptosis. Interestingly, methylenebis increased the level of LC3-II and belotecan independently decreased the level of p62, suggesting that methylenebis and belotecan target different steps of autophagy. Finally, we searched for compounds that are structurally similar to methylenebis. Our results imply that the specific structure of methylenebis contributes to its ability to activate autophagy.

## INTRODUCTION

Autophagy generally refers to macroautophagy, a process by which target molecules are delivered to autophagosomes. These autophagosomes subsequently fuse with lysosomes, where their contents are degraded [1]. This degradation is defined as autophagic flux; several methods have been developed to measure this process [1, 2]. Autophagy can be easily monitored by fluorescent microscopy and Western blot. Phosphatidyl ethanolamine (PE)-conjugated LC3 (LC3-II) indicates the formation of autophagosomes, and p62, a ubiquitin binding scaffold protein, is a useful marker to monitor the autophagy [1, 3]. Autophagy acts as a double-edged sword in the context of cancer because it can promote or inhibit cancer progression [4] [5]. Moreover, autophagy affects cancer cell responses to anticancer drug treatments and radiotherapy [6, 7]. For instance, cytotoxic autophagy can sensitize cancer cells to anticancer drugs, whereas cytoprotective autophagy can protect cancer cells from anticancer drugs [8, 9]. In addition, autophagy can have no effect on cancer therapy (“nonprotective” autophagy) [10, 11]. Several autophagy-regulating small molecules, such as chloroquine, are being investigated for their abilities to sensitize tumor cells to anticancer drugs [5, 12, 13]. However, there is no method for accurately predicting whether autophagy regulation sensitizes or protects tumor cells from a given anticancer drug [5].

Camptothecin is an alkaloid isolated from the Chinese tree *Camptotheca acuminata* that specifically targets DNA topoisomerase I [14]. Three derivatives of camptothecin (topotecan, irinotecan, and belotecan) are currently prescribed as anticancer drugs [15, 16]. Belotecan is effective in treating many cancers, including small cell lung cancer and ovarian cancer [17, 18]. Camptothecin has been reported to induce autophagosome formation in breast cancer cells and to induce cytoprotective autophagy, which delays apoptotic cell death [19]. Moreover, knockdown of autophagy related protein 5 (ATG5) and chloroquine treatment both enhance the cytotoxicity of camptothecin and its derivatives [20, 21]. On the contrary, micro RNA (miR-15a and mirR-16) induces autophagy and enhances the antitumor effect of camptothecin [22]. Therefore, combinatorial treatment of camptothecin or its derivatives with an additional autophagy-regulating small molecule is a potential approach for sensitizing tumor cells to anticancer drugs.

In this report, we demonstrate that 2,2′-methylenebis (6-*tert*-butyl-4-methylphenol), hereafter referred to as methylenebis, and its structurally related compounds regulate autophagy. Moreover, treatment of tumor cells with methylenebis sensitizes them to belotecan, a camptothecin derivative. Our results imply that belotecan and methylenebis have synergistic effects in cancer treatment.

## RESULTS

### Methylenebis regulates autophagy

**Figure 1:**
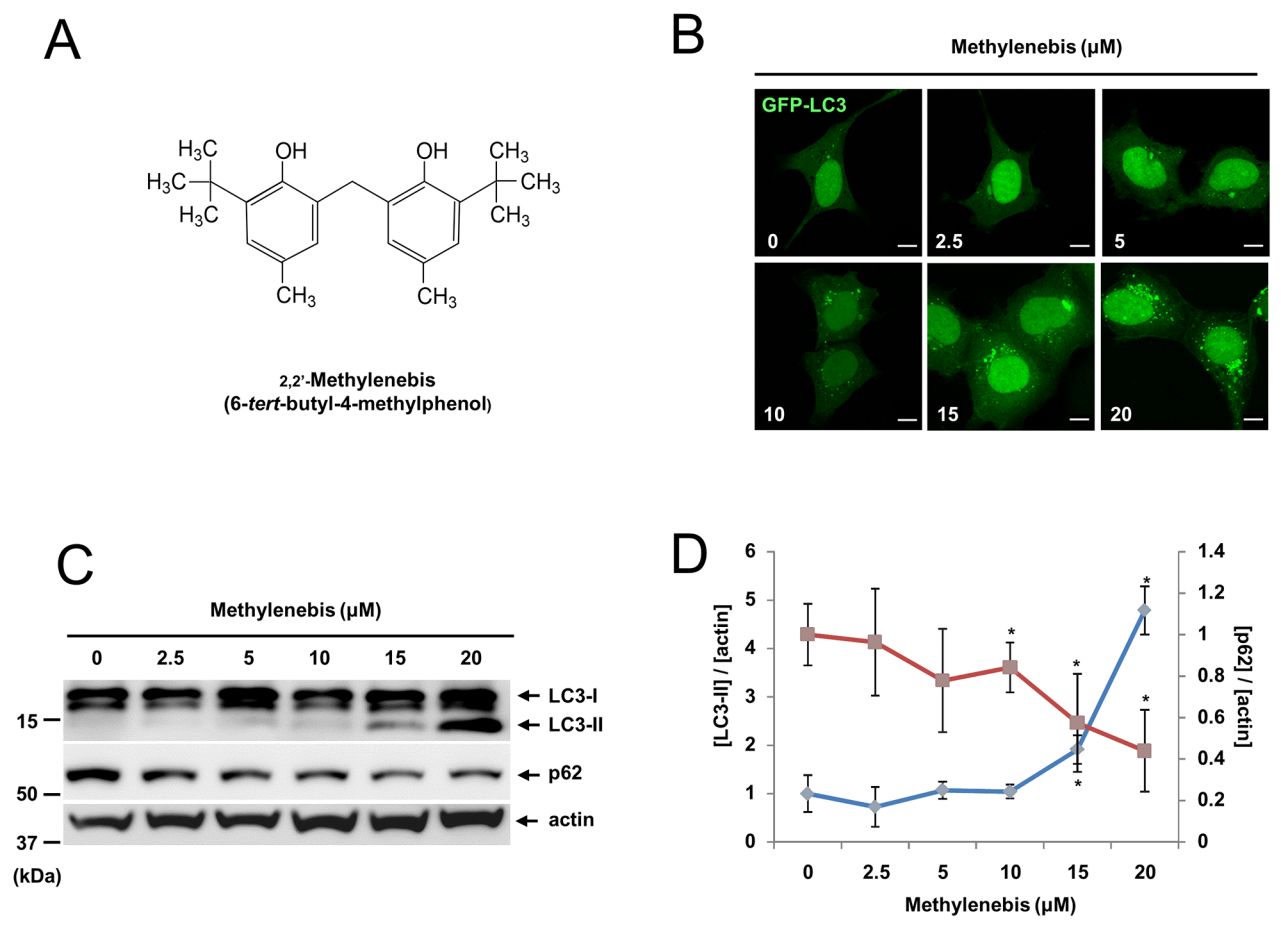
Methylenebis regulates autophagy **(A)** Chemical structure of methylenebis. **(B)** Methylenebis treatment induces autophagosome formation in HEK293 cells stably expressing GFP-LC3 (GFP-LC3 cells). GFP-LC3 cells were incubated with the indicated concentration of methylenebis for 24 h, after which they were fixed and analyzed with a confocal microscope. Bars: 10 μm. **(C)** Methylenebis treatment increased the level of LC3-II and decreased the level of p62. HEK293A cells were incubated with the indicated concentration of methylenebis (0, 2.5, 5, 10, 15, or 20 μM) for 24 h, after which cell lysates were generated and subjected to Western blotting with anti-LC3 antibodies and anti-p62 antibodies. **(D)** The LC3-II and p62 bands were quantified, and the relative expression levels are shown in the graph. Mock vs. methylenebis treatment, ^*^P < 0.05.

Previous studies have shown that autophagy is important for tumor cell survival. Moreover, excessive autophagy and autophagy deficiency often result in tumor cell death [23]. To identify novel compounds that regulate autophagy, we treated HEK293T cells stably expressing GFP-LC3 (GFP-LC3 cells) with a natural compound library and examined whether GFP-LC3 puncta were formed using a fluorescence microscope [24, 25]. We identified several compounds that regulate autophagy, of which 2,2′-methylenebis (6-*tert*-butyl-4-methylphenol) was one (Figure 1A). When we treated GFP-LC3 cells with methylenebis, we found that methylenebis induced autophagosome formation in a dose-dependent manner (Figure 1B). We also examined the levels of LC3-II and p62 in HEK293A cells. We found that the level of LC3-II was increased, whereas the level of p62 was decreased, indicating that autophagy is regulated by methylenebis (Figure 1C and 1D).

### Methylenebis activates autophagic flux

**Figure 2:**
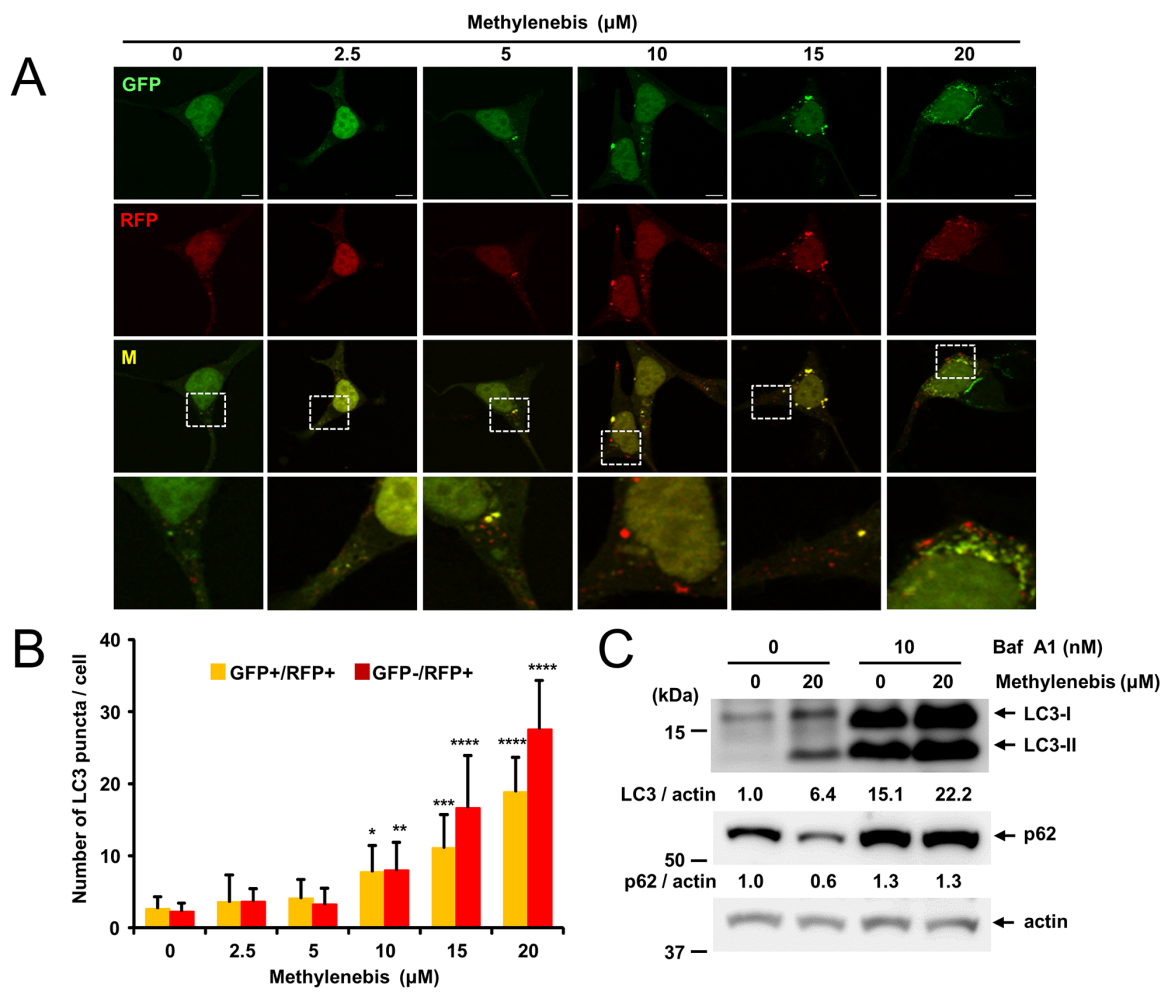
Methylenebis treatment induces autolysosome formation **(A)** Autophagosome and autolysosome formation induced by methylenebis. HEK293 cells were transfected with a plasmid encoding mRFP-GFP-LC3 and incubated for 24 h, after which they were treated with methylenebis for 24 h. Cells were analyzed with a confocal microscope. **(B)** Quantification of autophagosomal LC3 puncta (GFP+/RFP+) and autolysosomal LC3 puncta (GFP-/RFP+) (n=10). Control cells vs. methylenebis treatment, ^*^: P<0.005, ^**^: P<0.001, ^***^: P<0.0005, ^****^: P<0.0001. **(C)** Methylenebis treatment induces autophagic flux. MCF-7 cells were mock treated or treated with methylenebis (20 μM) in the presence or absence of bafilomycin A1 (10 nM).

Since autophagosomes can be formed by the activation of autophagy as well as by blocking autophagic flux, we next determined whether methylenebis activates or inhibits autophagic flux. The GFP-mRFP-LC3 plasmid (ptf-LC3) is commonly used to examine autophagic flux. Using this plasmid, the formation of red puncta (autolysosomes) and yellow puncta (autophagosomes) indicates the activation of autophagic flux [26]. Methylenebis treatment induced red puncta, suggesting that autolysosomes were formed (Figure 2A and 2B). We also examined the levels of LC3-II and p62 in response to methylenebis and bafilomycin A treatment. Methylenebis increased the levels of LC3-I and LC3-II. Similarly, bafilomycin A-induced blockade of autophagic flux also increased the levels of LC3-I and LC3-II (Figure 2C). We also examined the level of p62 after methylenebis and bafilomycin A treatment. Methylenebis decreased the level of p62, whereas bafilomycin A treatment inhibited this decrease (Figure 2C). These results collectively indicate that methylenebis activates autophagic flux.

### Synergistic antitumor efficacy of methylenebis and belotecan

**Figure 3:**
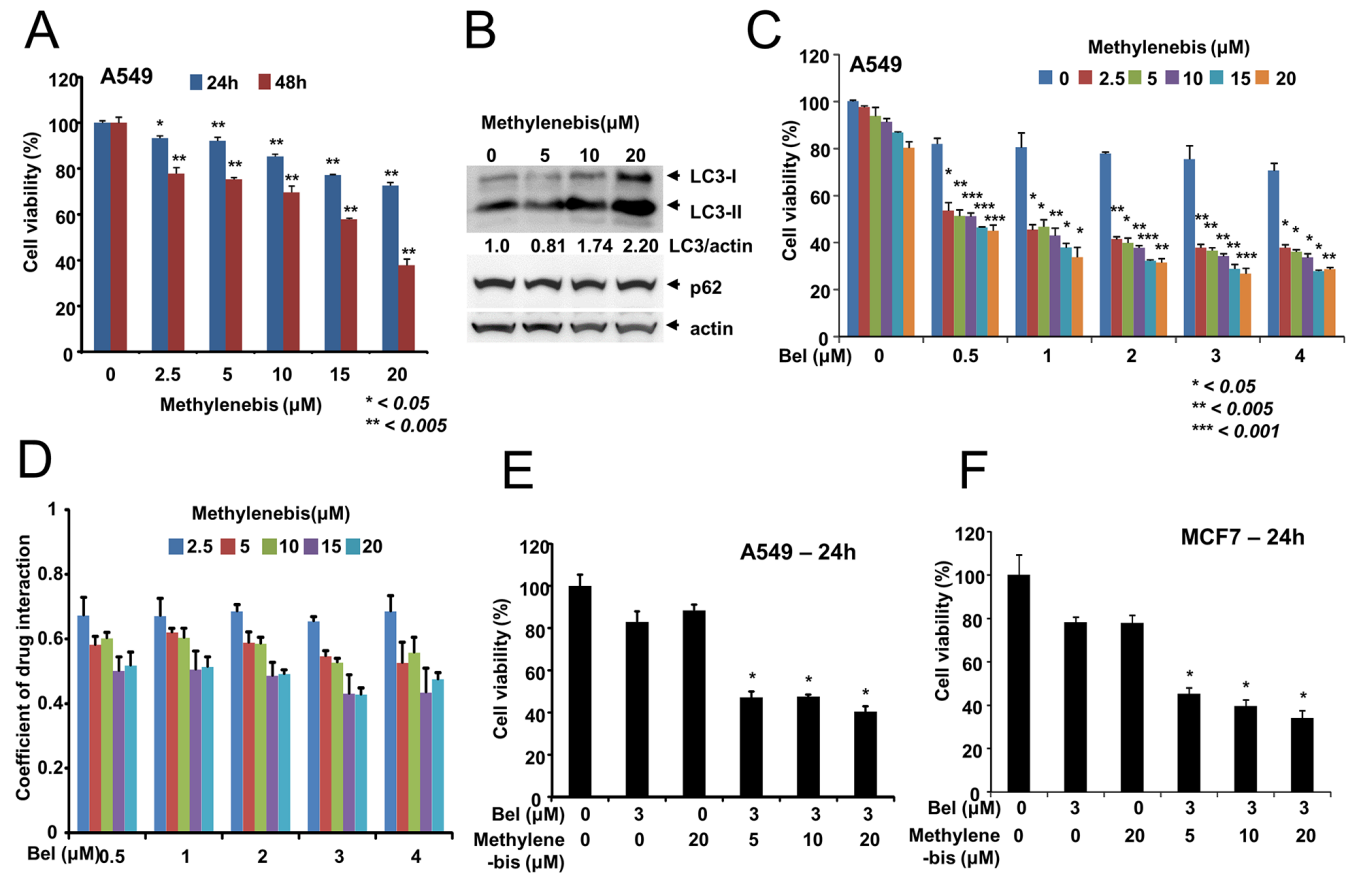
Synergistic antitumor efficacy of methylenebis and belotecan **(A)** Methylenebis induces cell death. A549 cells were incubated with the indicated concentration of methylenebis for 24 h or 48 h. Cell viability was measured using the MTT assay. Mock vs. drug treatment, ^*^, *P* < 0.05, ^**^ *P* < 0.005. **(B)** Methylenebis increases the level of LC3-II. A549 cells were treated with the indicated concentration of methylenebis for 24 h, after which cell lysates were generated and probed with anti-LC3 antibodies and anti-p62 antibodies. **(C)** The combination of methylenebis and belotecan synergistically enhances tumor cell death. A549 cells were cotreated with the indicated concentrations of methylenebis and belotecan. Belotecan treatment vs combined treatment, ^*^ *P* < 0.05, ^**^ *P* < 0.005, ^***^ *P* < 0.001. **(D)** The coefficient of drug interaction (CDI) was calculated and is shown in (C). **(E, F)** Synergistic cytotoxicity of methylenebis and belotecan in A549 and MCF-7 cells. Belotecan treatment vs. combined treatment, ^*^ *P* < 0.005.

During the screen, we observed that methylenebis treatment caused cell death (data not shown). Thus, we next examined whether methylenebis induces cell death. To this end, A549 non-small cell lung cancer cells were treated with methylenebis for 24 h or 48 h, after which cell viability was measured. Methylenebis induced tumor cell death in a dose-dependent manner (Figure 3A). We also examined whether methylenebis regulates autophagy in A549 cells. The results showed that the level of LC3-II in A549 cells was increased by methylenebis treatment, whereas the level of p62 was unchanged (Figure 3B). We used the GFP-mRFP-LC3 plasmid to examine the autophagic flux in A549 cells, and methylenebis treatment induced red puncta (autolysosmes) in A549 cells, indicates the activation of autophagic flux (Supplementary Figure 2). Autophagy regulators such as chloroquine can sensitize tumor cells to antitumor drugs [5]. Therefore, we next examined whether methylenebis can sensitize tumor cells to antitumor drugs. We tested several antitumor drugs and found that methylenebis sensitizes lung tumor cells to belotecan, a camptothecin-derived topoisomerase I inhibitor [17]. We next treated cells with combinations of belotecan (0, 0.5, 1, 2, 3, and 4 μM) and methylenebis (0, 2.5, 5, 10, 15, and 20 μM) and found that methylenebis enhanced belotecan-mediated tumor cell death (Figure 3C, Supplementary Figure 1). In addition, the coefficient of drug interaction (CDI) of methylenebis and belotecan was calculated to be less than 0.7 (Figure 3D). We also found that methylenebis enhanced belotecan-mediated cell death of MCF7 breast cancer cells (Figure 3E and 3F). When methylenebis was treated to MRC-5, a fetal lung fibroblast cell line, methylenebis showed the cytotoxicity to MRC-5, however methylenebis did not enhance belotecan mediated cell death of MRC-5 (Supplementary Figure 3). These results collectively indicate that methylenebis treatment sensitizes tumor cells to belotecan.

### Methylenebis and belotecan induce apoptosis of tumor cells

**Figure 4:**
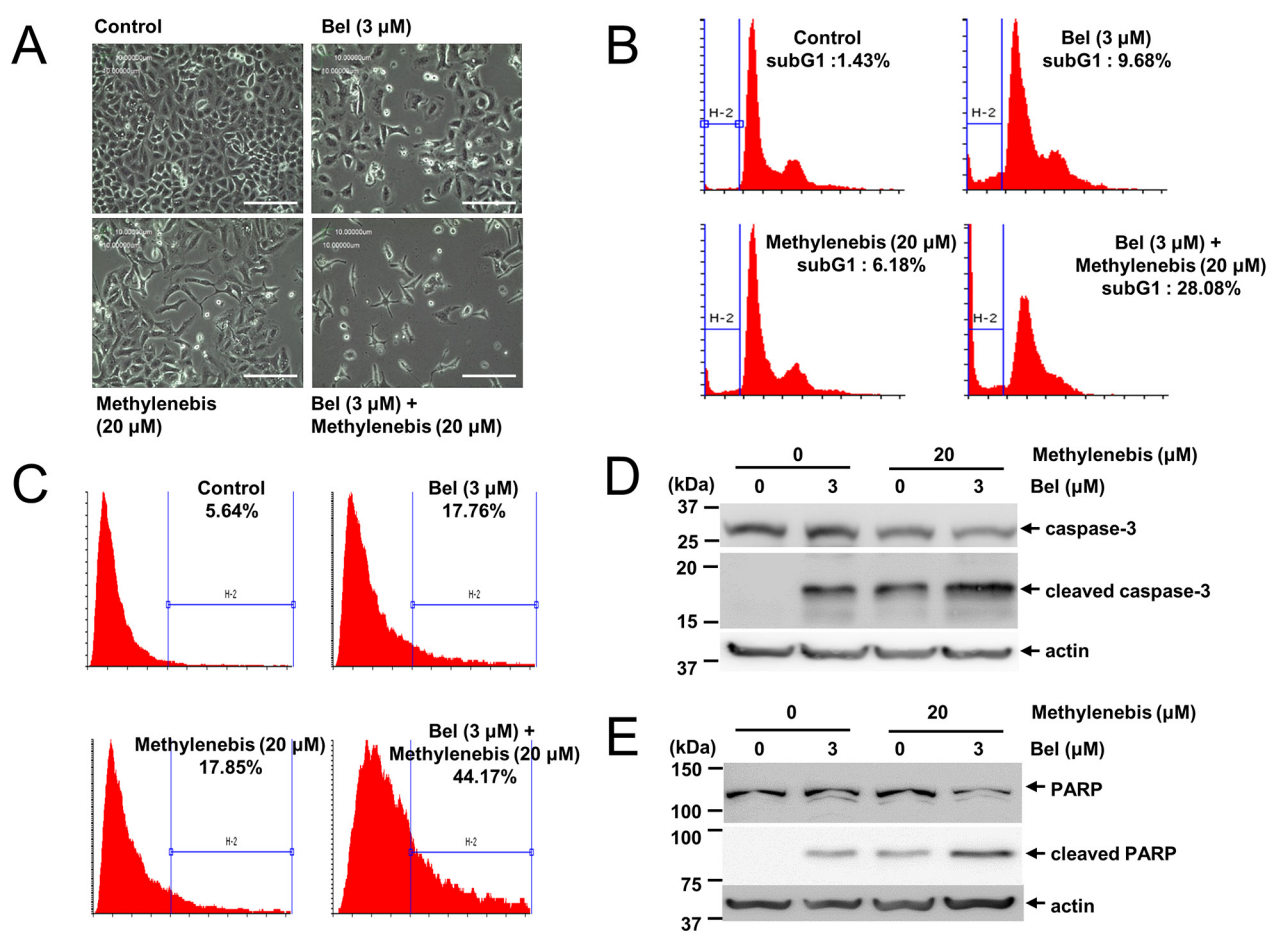
Methylenebis and belotecan induce apoptosis **(A)** Death of A549 cells treated with methylenebis and belotecan. Images were acquired with a light microscope (100X). Bars: 50 μm. **(B)** Apoptosis induction by methylenebis and belotecan. A549 cells were incubated with methylenebis and belotecan for 24 h. Cells were stained with propidium iodide and analyzed by flow cytometry. Percentages shown indicate the proportions of cells in sub-G1 phase. **(C)** Cells were stained with annexin V-FITC and analyzed by flow cytometry. Percentages shown indicate the annexin V positive cells. **(D, E)** A549 cells were incubated with methylenebis and belotecan for 24 h, and cell lysates were generated and subjected to Western blotting with the indicated antibodies.

Since methylenebis and belotecan treatment decreased the viability of tumor cells, we examined whether these drugs decrease cell viability by inducing apoptosis. We treated cells with either methylenebis or belotecan and observed the resultant cell death using a microscope. The results showed that cell death was increased by belotecan in combination with methylenebis (Figure 4A). Next, we examined cell death by flow cytometry. Methylenebis treatment and belotecan treatment each increased the sub-G1 population (apoptotic cells) by less than 10%, whereas methylenebis in combination with belotecan increased the sub-G1 population by up to 28% (Figure 4B). We further examined the apoptosis induction by annexin V assay. Methylenebis treatment and belotecan treatment each increased the annexin V positive cells (apoptosic cells) by less than 20%, whereas methylenebis in combination with belotecan increased the annexin V positive cells by up to 45% (Figure 4C). Finally, we confirmed the apoptosis induction by analyzing PARP cleavage and caspase-3 cleavage. While the cleaved PARP and cleaved caspase-3 were increased by methylenebis treatment and belotecan treatment each, both the cleaved PARP and cleaved caspase-3 were further increased by methylenebis in combination with belotecan (Figure 4D and Figure 4E). These findings collectively indicate that methylenebis and belotecan decrease cell viability by inducing apoptosis.

### Methylenebis and belotecan regulate autophagy separately

**Figure 5:**
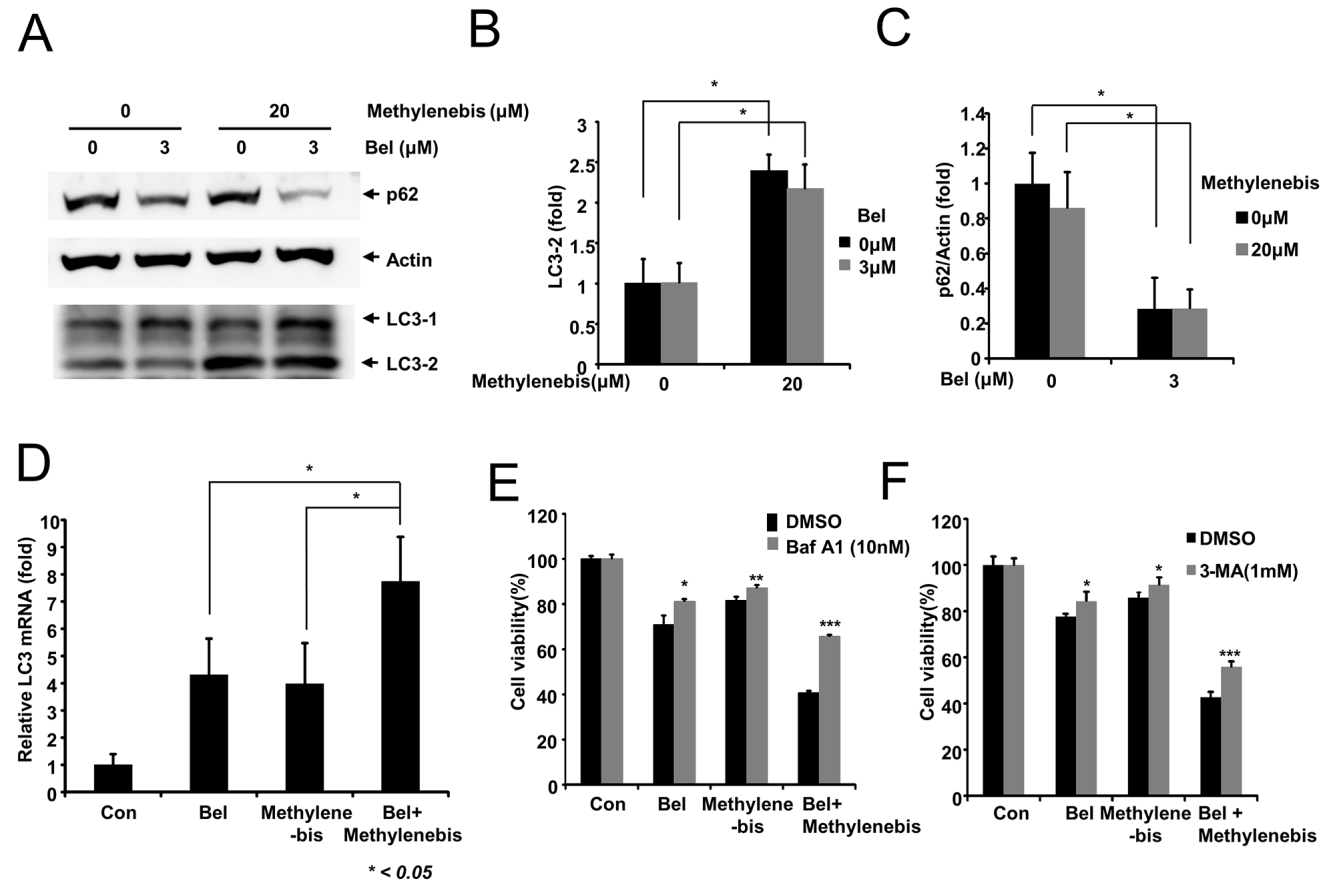
Regulation of autophagy by methylenebis and belotecan **(A)** Methylenebis and belotecan regulate autophagy. A549 cells were treated with methylenebis and belotecan for 24 h, after which cell lysates were generated and probed with the indicated antibodies. **(B, C)** Methylenebis increases the level of LC3-II, and belotecan decreases the protein level of p62. The LC3-II and p62 bands were quantified; relative expression levels are shown. Control vs. drug treated, ^*^ *P* < 0.05. **(D)** Methylenebis and belotecan induce expression of LC3 mRNA. A549 cells were treated with methylenebis and belotecan for 24 h, after which the expression of LC3 mRNA was measured by quantitative real-time PCR. Single drug treatment vs. combined treatment, ^*^, *P* < 0.05. (E, F) Treatment with an autophagy inhibitor attenuates cell death induced by the combination of belotecan and methylenebis. A549 cells were treated with methylenebis and belotecan for 24 h, after which they were treated with bafilomycin A1 **(E)** or 3-methyladenine **(F)**. Control vs. Baf A1 or 3-MA treated, ^*^ *P* < 0.05, ^**^ *P* < 0.005, ^***^ *P* < 0.001.

Since methylenebis induces autophagy by increasing the level of LC3-II, we examined the regulation of autophagy by methylenebis and belotecan. Belotecan is a derivative of camptothecin, which has been reported to induce autophagy [27]. Similar to camptothecin, belotecan induced autophagy by decreasing the level of p62, whereas methylenebis treatment did not change the level of p62 (Figure 5A and 5C). Like belotecan, methylenebis increased the level of LC3-II; methylenebis-mediated elevation of the level of LC3-II was not affected by cotreatment with belotecan (Figure 5A and 5B). Therefore, treatment with methylenebis and belotecan increased the LC3-II level and decreased the p62 level (Figure 5A). These results suggest that methylenebis and belotecan regulate separate steps of autophagy.

Autophagy inducers often increase the expression of LC3 mRNA; thus, we examined the mRNA level of LC3 upon methylenebis and belotecan treatment. Methylenebis treatment and belotecan treatment each upregulated LC3 mRNA expression in a dose-dependent manner (Supplementary Figure 4); moreover, LC3 mRNA was expressed significantly higher in cells cotreated with methylenebis and belotecan compared to cells treated with only one of the drugs (Figure 5D). These results indicate that methylenebis enhances belotecan-mediated induction of autophagy.

Finally, we examined whether autophagy inhibitors suppress the antitumor efficacy of belotecan and methylenebis. A549 cells were treated with DMSO, belotecan, methylenebis, or belotecan/methylenebis for 24 h, after which they were treated with bafilomycin A1 and 3-methyladenine. Bafilomycin A1 and 3-MA significantly inhibited cell death, indicating that autophagy regulation is involved in methylenebis- and belotecan-induced cell death (Figure 5E and 5F).

### Methylenebis and belotecan treatment inhibits the growth of A549 xenografts in nude mice

**Figure 6:**
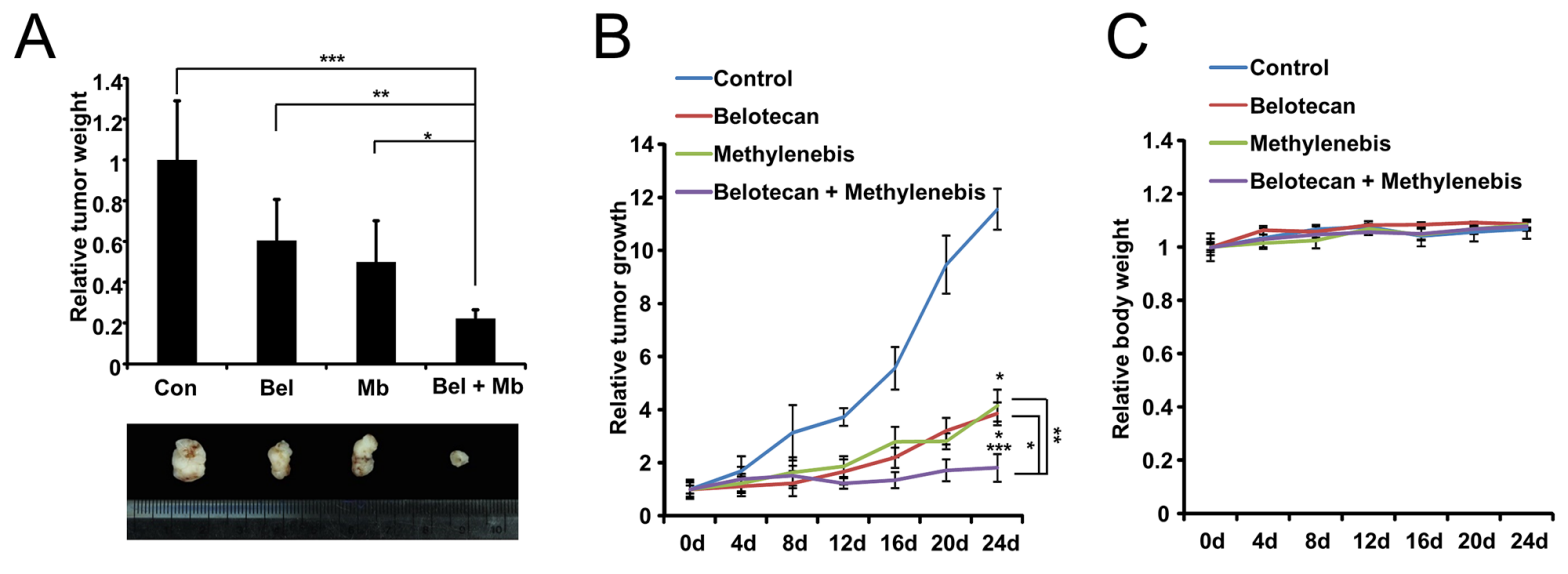
Methylenebis enhances the antitumor efficacy of belotecan in a xenograft mouse model **(A)** Weights of excised tumors at the end of the experiment (n=6). Athymic nude mice were first injected with A549 cells (1×10^7^ cells) and then injected intratumorally with methylenebis and belotecan every 4 days. Tumor weight, ^*^ *P* < 0.05, ^**^ *P* < 0.01, ^***^ *P* < 0.005. **(B)** Growth curve of xenograft tumors treated with PBS, methylenebis, belotecan, or methylenebis/belotecan. Data are expressed as mean±SD. Tumor size, ^*^ *P* < 0.01, ^**^ *P* < 0.005, ^***^ *P* < 0.0005. **(C)** The relative body weight change during the experiment.

We next investigated whether the combined methylenebis and belotecan treatment affects tumor growth. Nude mice were injected in both flanks with A549 cells. When equally sized tumors were formed, animals were treated intratumorally with PBS, methylenebis, belotecan, or methylenebis/belotecan every 4 days. The tumor volumes and tumor weights of the sacrificed mice were measured to compare the efficacy of the anticancer drugs. Methylenebis treatment and belotecan treatment each resulted in significantly reduced tumor growth, while cotreatment with methylenebis and belotecan resulted in additional reduction (Figure 6A and 6B). In addition, the average body weight of control or drug treated mice did not vary significantly (Figure 6C). These results show that methylenebis enhances the antitumor effect of belotecan *in vivo*.

### Methylenebis and structurally related compounds show similar effects

**Figure 7:**
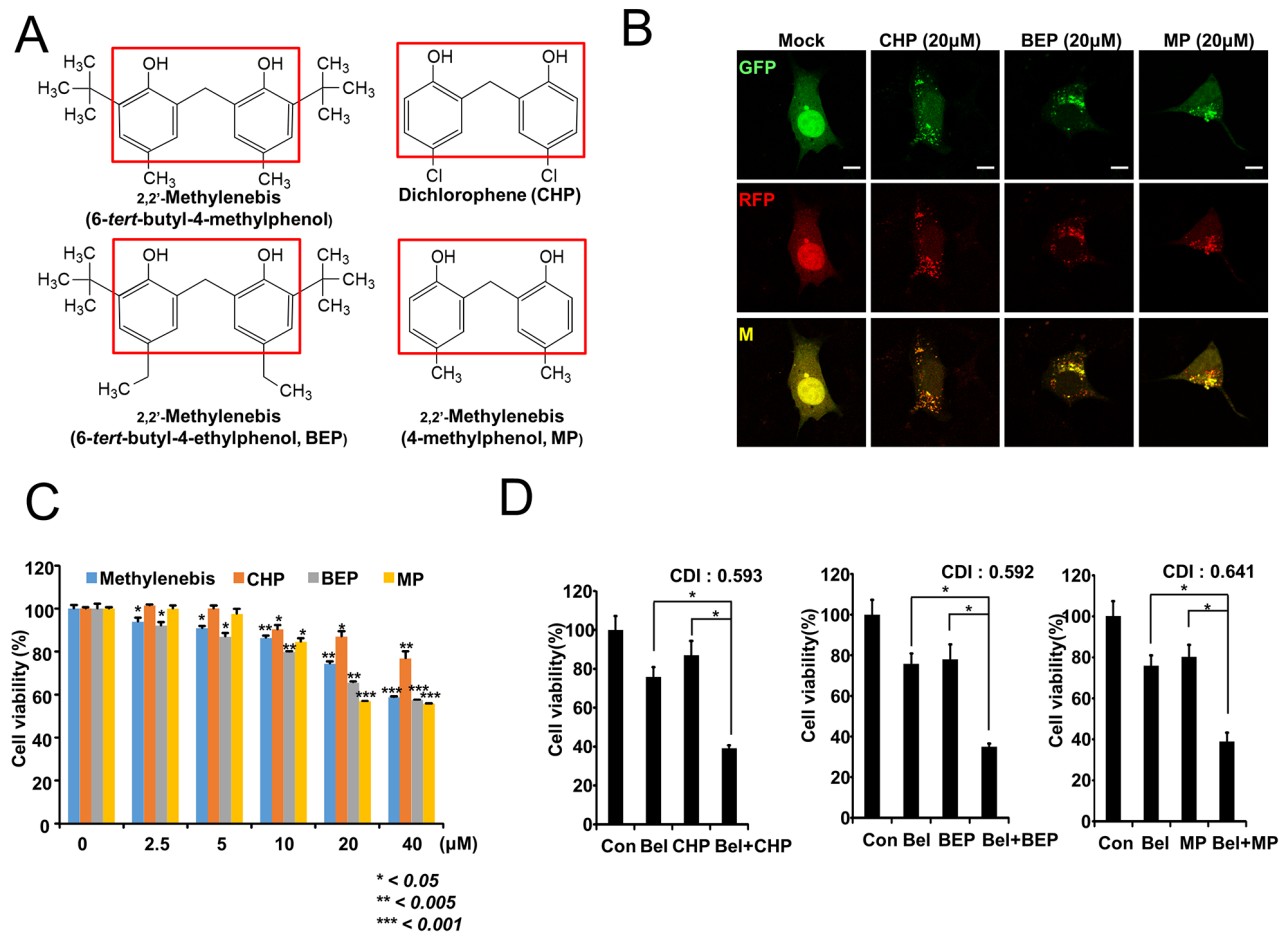
Methylenebis and structurally related chemicals have similar antitumor efficacy **(A)** Chemical structures of methylenebis-like chemicals that have similar antitumor efficacy. **(B)** Autophagosome and autolysosome formation after treatment with CHP, BEP, or MP. Bars: 10 μm. **(C)** Viability of A549 cells treated with CHP, BEP, or MP. **(D)** Antitumor efficacy enhancement of belotecan by CHP, BEP, and MP. Single drug treatment vs. combined treatment, ^*^ *P* < 0.005.

Since methylenebis showed synergistic antitumor effects with belotecan, we examined the antitumor effects of compounds structurally related to methylenebis. First, we examined the ability of structurally related compounds to regulate autophagy. We found that methylenebis, dichlorophene (CHP), 2,2′-methylenebis (6-*tert*-butyl-4-ethylphenol, BEP), and 2,2′-methylenebis (4-methylphenol, MP) had similar activity (Figure 7A, 7B and Supplementary Figure 5). Each of these compounds has two phenol rings with two hydroxyl groups. We also examined the viability of A549 cells treated with these compounds and observed that all compounds had similar cytotoxic activity (Figure 7C). Finally, we examined the synergistic antitumor effects of CHP, BEP, and MP. The CDI values were all less than 1, indicating that these compounds synergistically enhance the antitumor effect of belotecan, similar to methylenebis (Figure 7D).

## DISCUSSION

Autophagy regulation is involved in tumor cell survival. Both activation and inhibition of autophagy can enhance the antitumor efficacy of anticancer drugs that are clinically available. Therefore, the discovery of novel small molecules that regulate autophagy can help enhance existing cancer therapies. We thus searched for novel small molecules that regulate autophagy and found that methylenebis is an autophagy-regulating small molecule.

Previous studies have shown that 2,2′-methylenebis (6-*tert*-butyl-4-methylphenol, methylenebis) and 2,2′-methylenebis (6-*tert*-butyl-4-ethylphenol, BEP) are antioxidants and potentially toxic to rats [28, 29]. The LD50 of 2,2′-methylenebis (6-*tert*-butyl-4-methylphenol, methylenebis) is 5g/kg, and the no-observed-adverse-effect level (NOAEL) is 12.7 mg/kg [29]. Similarly, the NOAEL of 2,2′-methylenebis (6-*tert*-butyl-4-ethylphenol, BEP) is 12 mg/kg [28]. We used methylenebis (3.4 mg/kg) for the tumor xenograft experiment. The dose we used is much lower than the reported NOAEL dose. In addition, none of the mice died as a result of the methylenebis injection. Further safety experiments should be performed to realize the potential of methylenebis as an anticancer drug.

We used GFP-LC3 cells to screen for autophagy-regulating compounds. In these cells, autophagosomes can be clearly identified with a fluorescence microscope. However, the formation of autophagosomes can be induced by activation or inhibition of autophagic flux. We investigated whether methylenebis activates or inhibits autophagic flux. Methylenebis treatment induced autolysosome formation and decreased the p62 level (Figure 1C), suggesting that methylenebis activates autophagic flux. Of particular note, we found that methylenebis treatment increased the protein level of LC3-II as well as the mRNA level of LC3 (Figure 5D). The accumulation of LC3-II can contribute to the formation of autophagosomes, an early step in autophagy. Although we did not identify the direct target of methylenebis for autophagy regulation, methylenebis appears to be involved in autophagy by regulating the level of LC3.

Interestingly, belotecan regulates autophagy by decreasing the level of p62, a protein that is involved in later steps of autophagy, whereas methylenebis regulates autophagy by increasing LC3-II, which is involved in an early step of autophagy. Therefore, the mechanisms of autophagy regulation by belotecan and methylenebis appear to be independent of each other. Belotecan treatment did not affect the methylenebis-mediated increase of LC3-II, and methylenebis did not affect the belotecan-mediated decrease of p62. Since belotecan and methylenebis target different stages of autophagy, belotecan and methylenebis can synergistically increase autophagic flux.

To the best of our knowledge, this is the first report that belotecan regulates autophagy. Belotecan is a derivative of camptothecin, which has been reported to induce cytoprotective autophagy. Therefore, knockdown of ATG5 and chloroquine treatment sensitize tumor cells to camptothecin and to camptothecin-related compounds [20, 21]. Since we showed that methylenebis increases autophagy in this study, it is seemingly paradoxical that the promotion of autophagy sensitizes tumor cells to belotecan. However, autophagy is a double-edged sword, and the proper level of autophagy is important for cell survival. Imbalance of autophagy activity is believed to contribute to cell death. For example, cytoprotective autophagy is induced for cells to respond to anticancer drugs, and the deregulation of cytoprotective autophagy by additional compounds can sensitize tumor cells to the drug treatment. However, it is also possible that belotecan or methylenebis induces cytotoxic autophagy, and that the excessive autophagic flux induced by these two compounds contributes to the enhanced cell death [22].

The enhancement of belotecan activity by methylenebis could help overcome drug resistance in cancer cells and could also decrease the side effects of anticancer drugs by minimizing their doses. Since we identified the initial compound and later found that structurally related compounds had similar activity, there might be additional structurally related compounds that have fewer side effects and improved efficacy. Further studies are required to move the current findings forward into preclinical or clinical trials.

## MATERIALS AND METHODS

### Cell culture and cell viability assay

HEK293T, A549, and MCF7 cells were grown in DMEM medium (Welgene, Korea) supplemented with 10% fetal bovine serum (Gibco, Waltham, MA, USA) and 1% antibiotic-antimycotic solution (Welgene, Seoul, Korea). Cell viability was measured using the [4,5-dimethylthiazol-2-yl]-2,5-diphenyltrazolium bromide (MTT) assay. Briefly, cells were seeded in a 24-well plate, incubated overnight, and treated with the antitumor drugs. At the indicated time, MTT solution was added to a final concentration of 1 mg/ml, and the mixture was incubated for an additional 3 hours. MTT was purchased from USB Corporation (Cleveland, OH, USA). Methylenebis was obtained from the Korea Bioactive Natural Material Bank (KBNMB) and from Sigma (St. Louis, MO, USA). For cell cycle analysis, cells were washed and fixed with 70% ethanol. After centrifugation, cells were resuspended in PBS containing 0.25 mg/ml propidium iodide (PI) and 10 mg/ml RNase A (Sigma, St. Louis, MO, USA). Cells were analyzed on a FACSCalibur flow cytometer (Becton-Dickinson, Mountain View, CA, USA). At least 10,000 events per sample were analyzed with Flowing software (Turku University, Finland). For annexin V assay, cells were incubated with FITC conjugated annexin V (KOMA Biotechnology, Seoul, Korea). A549 cells were detached from the plate and incubated with annexin V-FITC for 10 min. The stained cells were analyzed in a FACSCalibur (10,000 cells / sample).

### Western blotting

For Western blot analysis, polypeptides in whole cell lysates were resolved by SDS-PAGE and transferred to NC or PVDF membrane filters. Proteins were detected with primary antibodies (1:1000 or 1:5000 dilution) using an enhanced chemiluminescence (ECL) system. Images were acquired using a Chemidoc-it 410 imaging system (UVP, Upland, CA) and an LAS4000 system (GE Healthcare, Uppsala, Sweden). The following primary antibodies were used: anti-LC3 (MBL International, Watertown, MA, USA), anti-p62 (MBL International), anti-caspase 3 (Cell Signaling Technology, Beverly, MA, USA), anti-cleaved caspase 3 (Cell Signaling Technology), anti-PARP-1 (Santa Cruz Biotechnology, Santa Cruz, CA, USA), anti-cleaved PARP (Genetex, San Antonio, TX, USA) and anti-actin (ABM, Richmond, BC, Canada).

### Immunofluorescence and confocal microscopy

HEK293T cells were grown on sterilized glass coverslips. After plasmid transfection and drug treatment, cells were fixed with 4% paraformaldehyde. The slides were washed 3 times with PBS and subsequently mounted in mounting medium containing DAPI (Vector Laboratories, Burlingame, CA, USA). Images were captured with a Carl Zeiss LSM710 confocal microscope (Oberkochen, Germany). pTF-LC3 was purchased from Addgene (Cambridge, MA, USA).

### Quantitative real-time PCR

Total RNA from each sample was extracted using Trizol reagent (Invitrogen, Carlsbad, CA, USA). Reverse transcription was carried out with an M-MLV RT kit (Enzynomics, Daejeon, South Korea) according to the manufacturer’s protocol. The following primers were used for amplification: LC3, forward (ACCATGCCGTCGGAGAAG) and reverse (ATCGTTCTATTATCACCGGGATTTT); RPL4, forward (GCTCTGGCCAGGGTGCTTTTG) and reverse (ATGGCGTATCGTTTTTGGGTTGT). Real-time PCR was performed with a Step One Plus Real-Time PCR system (ABI, Foster City, CA).

### Animal experiments

For the tumor xenograft experiments, 1×10^7^ A549 cells were injected subcutaneously into the hind limbs of nude mice. When the tumor size reached 100 mm^3^, methylenebis and belotecan were administered intratumorally every 4 days. After 4 weeks of treatment (belotecan, 0.65 mg/kg; methylenebis, 3.4 mg/kg), mice were sacrificed. Tumors were excised and fixed with 4% paraformaldehyde in PBS. All animal work was reviewed and approved by the Institutional Biosafety Committee - Institutional Animal Care and Use Committee (IBC-IACUC) of Yonsei University Wonju Campus (IACUC Approval Number: YWCI-201707-015-12).

### Analysis of drug interaction

The coefficient of drug interaction (CDI) was used to analyze the drug interaction between two different drugs. CDI is defined by the following formula; CDI = AB/(AxB) [30, 31]. According to each MTT absorbance, AB is the ratio of the two-drug combination group to the control group, and A or B is the ratio of the single drug group to the control group. CDI < 1 indicates synergism, CDI < 0.7 significant synergism, CDI = 1 additivity and CD > 1 antagonism [30].

## SUPPLEMENTARY MATERIALS FIGURES


