# Supplementary material for: 2,2′-Methylenebis (6-tert-butyl 4-methylphenol) enhances the antitumor efficacy of belotecan, a derivative of camptothecin, by inducing autophagy
Source: Oncotarget. 2017 Dec 1;8(70):115068–78. doi: 10.18632/oncotarget.22858 (PMC5777754; doi:10.18632/oncotarget.22858)
Supplement: Supplementary file 1 [file oncotarget-08-115068-s001.pdf]

2,2'-Methylenebis (6-tert-butyl 4-methylphenol) enhances the antitumor efficacy of belotecan, a derivative of camptothecin, by inducing autophagy

SUPPLEMENTARY MATERIALS

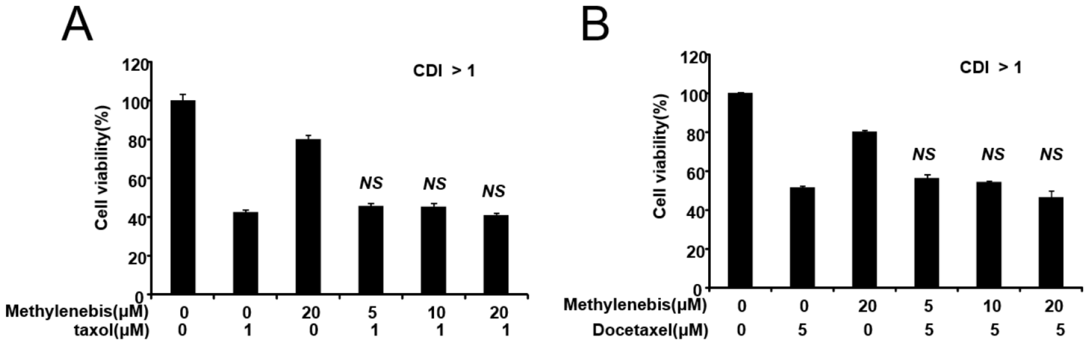

Supplementary Figure 1: Methylenebis does not enhance the antitumor efficacy of taxol or docetaxel. **(A)** Death of A549 cells treated with methylenebis and taxol. The coefficient of drug interaction was larger than 1. Taxol treatment vs. combined treatment, NS, not significant. **(B)** Death of A549 cells treated with methylenebis and docetaxel. The coefficient of drug interaction was larger than 1. Docetaxel treatment vs. combined treatment, NS, not significant.

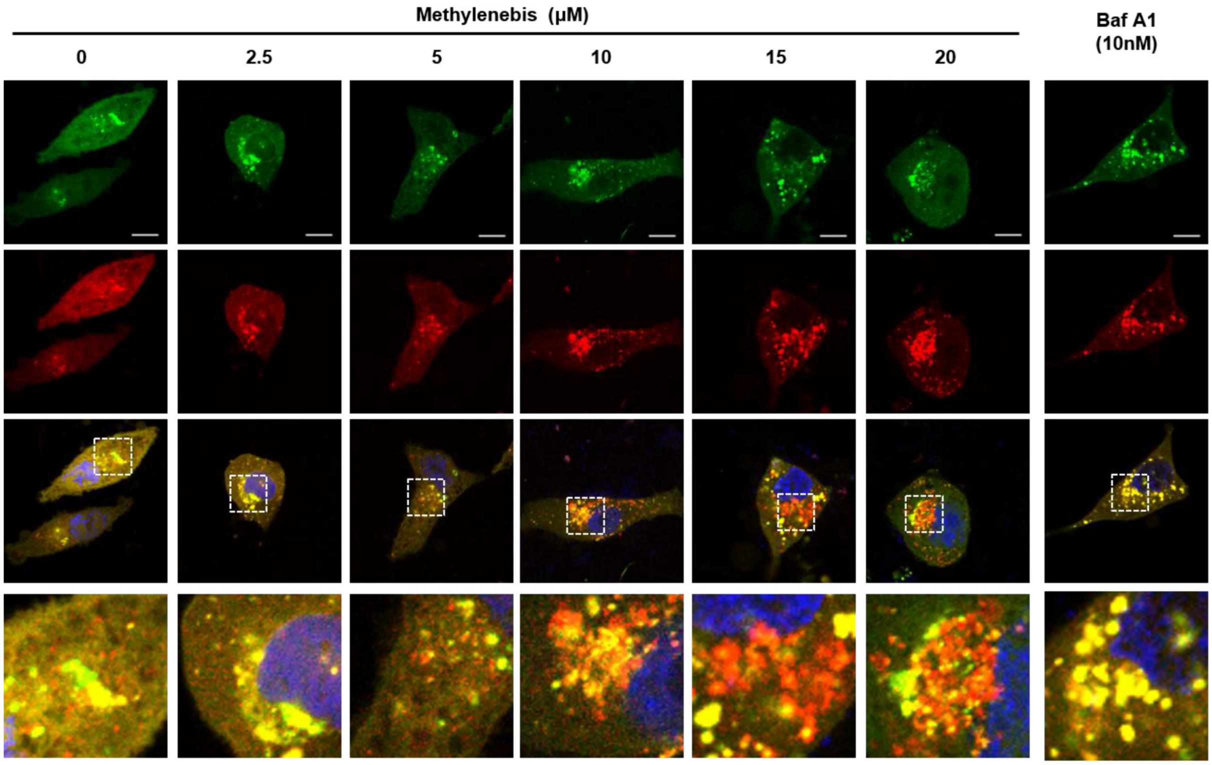

Supplementary Figure 2: Methylenebis treatment induces autolysosome formation. Autophagosome and autolysosome formation induced by methylenebis. A549 cells were transfected with a plasmid encoding mRFP-GFP-LC3. After 24 h, cells were treated with the indicated concentration of methylenebis or bafilomycin for 24 h. Cells were analyzed with a confocal microscope. Bafilomycin A1 treatment resulted in the formation of autophagosomes, but not autolysosomes.

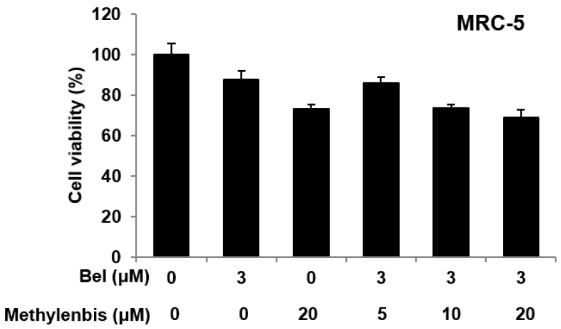

Supplementary Figure 3: Methylenebis does not enhance the antitumor efficacy of belotecan in normal cells. MRC-5 cells were treated with belotecan and methylenebis for 24h, and cell viability was measured using the MTT assay. The coefficient of drug interaction was larger than 1.

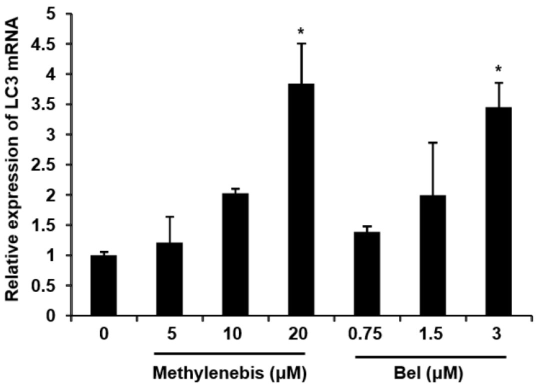

Supplementary Figure 4: Methylenebis and belotecan induce expression of LC3 mRNA in a dose-dependent manner. A549 cells were treated with either methylenebis or belotecan for 24 h, after which the expression level of LC3 mRNA was measured by quantitative real-time PCR. Mock vs. drug treatment, *, $P < 0.005$.

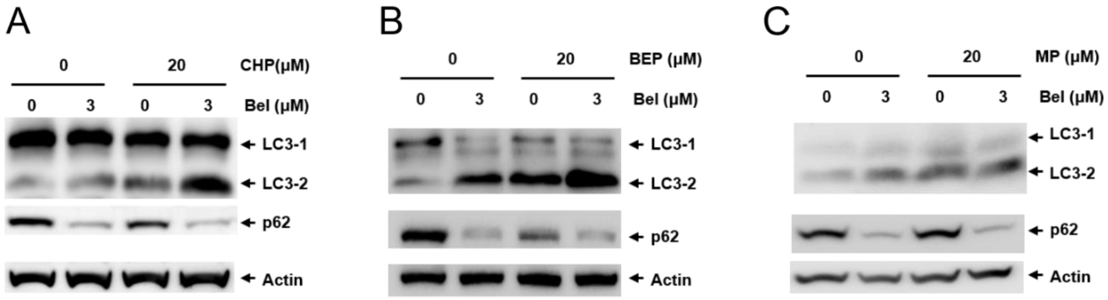

Supplementary Figure 5: Treatment of belotecan with compounds structurally similar to methylenebis regulates autophagy. (A) A549 cells were treated with CHP (20 μM) or belotecan (3 μM) for 24 h, after which cell lysates were generated and probed with the indicated antibodies. **(B)** A549 cells were treated with methylenebis-BEP (20 μM). **(C)** A549 cells were treated with methylenebis-MP (20 μM).
